# A 3D pooled PBMC-organoid co-culture platform for profiling immune susceptibility and PD-1 blockade response in gastric cancer

**DOI:** 10.1186/s12916-026-05006-4

**Published:** 2026-06-24

**Authors:** Han Byeol Mun, Woo Sun Kwon, Chan Hee Park, Harim Koo, Jinsoo Jang, Byeong Gyu Yoon, Juin Park, Ye Jin Moon, Choong-kun Lee, Hei-Cheul Jeung, Tae Soo Kim, Sun Young Rha

**Affiliations:** 1 Brain Korea 21 PLUS Project for Medical Science, Yonsei University College of Medicine, Seoul, South Korea; 2 Song-Dang Institute for Cancer Research, Yonsei University College of Medicine, Seoul, Republic of Korea; 3 Department of Medical Science Convergence, Graduate School of Medical Science, University of Ulsan, Ulsan, Republic of Korea; 4 Department of Medicine, Yonsei University College of Medicine, Seoul, Republic of Korea; 5 Division of Medical Oncology, Department of Internal Medicine, Yonsei Cancer Center, Yonsei University College of Medicine, Seoul, Republic of Korea; 6 Department of Medical Oncology, Gangnam Severance Hospital, Yonsei University College of Medicine, Seoul, Republic of Korea

**Keywords:** Gastric cancer, Peritoneal metastasis, Patient-derived organoids, Co-culture, Immunotherapy, Immune resistance

## Abstract

**Background:**

Peritoneal metastatic gastric cancer (PMGC) is associated with a dismal prognosis and limited benefit from immune checkpoint inhibitors (ICIs). Although PD-1/PD-L1 blockade has improved outcomes in selected patients, therapeutic responses remain highly heterogeneous, even among PD-L1-high tumors. The lack of preclinical models that functionally recapitulate tumor-immune interactions in a three-dimensional (3D) context has hindered the investigation of immune susceptibility and resistance mechanisms in PMGC.

**Methods:**

We established a standardized allogeneic 3D co-culture platform integrating patient-derived PMGC organoids with pooled peripheral blood mononuclear cells (PBMCs) from healthy donors. An optimized Matrigel-embedded configuration was used to enable sustained immune cell infiltration and tumor-immune contact. Immune-mediated cytotoxicity, organoid susceptibility phenotypes, and responses to PD-1 blockade with pembrolizumab were functionally assessed. Comparative proteomic profiling was performed, and differential expression between groups was evaluated using appropriate statistical tests with false discovery rate correction. Group comparisons were conducted using Student’s t-test.

**Results:**

The optimized 3D embedded system enabled dynamic infiltration of activated T cells into organoid structures. PMGC organoids exhibited heterogeneous susceptibility to immune-mediated cytotoxicity, classifying them into cytolytic and noncytolytic phenotypes independent of HLA mismatching. Comparative proteomic profiling revealed that cytolytic lines were enriched in metabolic pathways, whereas noncytolytic lines showed enrichment of immune-related signaling. Notably, response to pembrolizumab varied even among PD-L1-high organoids. Nonresponsive PD-L1-high lines were characterized by elevated baseline expression of alternative immune checkpoint ligands, specifically CD112 (TIGIT ligand) and galectin-9 (TIM-3 ligand).

**Conclusions:**

We established a robust pooled PBMC-organoid co-culture platform that enables functional assessment of tumor-immune dynamics in PMGCs. This system serves as a functional ex vivo tool for evaluating immunotherapy responses and for examining the expression patterns of alternative immune checkpoint ligands associated with differential PD-1 blockade response. These findings support the utility of this ex vivo PMGC co-culture system for evaluating heterogeneous responses to PD-1 blockade and associated baseline differences in alternative immune checkpoint ligand expression among PD-L1-high organoids.

**Supplementary Information:**

The online version contains supplementary material available at https://doi.org/10.1186/s12916-026-05006-4.

## Background

Gastric cancer (GC) remains the fifth leading cause of cancer-related mortality globally [1–3]. Despite advances in early detection and treatment strategies, the prognosis of patients with GC with peritoneal metastasis (PMGC) remains poor, with a 5-year survival rate of < 10% [4–6]. The therapeutic landscape of GC has been significantly reshaped by the introduction of immune checkpoint inhibitors (ICIs), specifically those targeting the programmed death-1 (PD-1) and programmed death ligand 1 (PD-L1) axes.

Although PD-1/PD-L1 inhibitors have improved outcomes in selected patients, the clinical benefit remains limited to patients with specific biomarkers, such as high microsatellite instability, high PD-L1 expression, Epstein-Barr virus (EBV) positivity, or high tumor mutational burden [7–12]. Consequently, there is an urgent need for preclinical models that can functionally model tumor-immune dynamics and identify the mechanisms underlying differential ICI responses.

Conventional two-dimensional (2D) culture systems have contributed to our understanding of cancer biology but fail to reproduce the three-dimensional (3D) structure, cellular heterogeneity, and stromal-tumor interactions present in primary tumors [13–16]. In contrast, patient-derived organoids (PDOs) serve as physiologically relevant 3D models that retain the genomic, histological, and functional characteristics of parental tumors [17–19]. Particularly in PMGC, organoids can be established from malignant ascites or pleural effusions obtained through minimally invasive procedures, offering a practical platform when solid tumor biopsies are unavailable [20, 21].

Although patient-derived organoids offer significant advantages, conventional organoid cultures lack immune components, limiting their use in assessing checkpoint inhibitor responses [22–25].

To overcome these limitations, several approaches have been used for organoid-immune cell co-cultures, including the use of autologous tumor-infiltrating lymphocytes, peripheral blood mononuclear cells (PBMCs), or specific immune cell subsets. Although autologous co-cultures best replicate the in vivo environment, they are often limited by tissue availability and immune cell yield [26–34]. In contrast, allogeneic approaches allow standardized immune inputs and scalability for experimental screening [35–38]. However, allogeneic models face challenges stemming from the lack of standardized immune engagement within 3D systems.

In this context, we established an allogeneic pooled PBMC-organoid co-culture platform as a functional assay system to evaluate immunotherapy responses and explore the mechanisms of immune resistance in PMGC, thereby addressing the current limitations of the existing models.

## Methods

### Patient samples

After written informed consent was obtained, malignant ascites or pleural effusion samples were collected from patients with GC. This study was approved by the Institutional Review Board of Severance Hospital, Yonsei University Health System (IRB approval number: 4-2014-0638). Human epidermal growth factor receptor 2 (HER2) status was determined by immunohistochemistry (IHC) using the HER2 4B5 antibody (Ventana Medical Systems, Tucson, AZ, USA), following the guidelines of the American Society of Clinical Oncology/College of American Pathologists. PD-L1 expression was assessed via the PD-L1 IHC 22C3 pharmDx assay (Agilent Technologies/Dako, Carpinteria, CA, USA) with combined positive score (CPS) calculations.

### Establishment of PMGC organoids

PMGC organoids were established from ascites or pleural effusion samples following the standard protocol developed by the Song-Dang Institute for Cancer Research (SICR), which is based on previously reported methods [14, 39]. The fluids were transferred into 50-mL tubes and washed with phosphate-buffered saline (PBS). The cell pellets were suspended in Matrigel (#356255; Corning, Glendale, AZ, USA) and plated as ten 10-µL droplets per well in 6-well plates. After polymerization at 37 °C for 15 min, the organoid medium was added, the medium was changed every 2–3 days, and the cells were passaged every 5–7 days. The cultured cells were incubated at 37 °C in a humidified atmosphere of 5% CO2. Organoids that expanded through ≥ 5 passages underwent short tandem repeat profiling using matched PBMC DNA to verify patient identity.

### Genomic profiling

For organoid characterization, whole-genome sequencing (WGS) was performed via the CancerVision platform (Inocras Inc., Daejeon, South Korea). Genomic DNA from PMGC organoids and matched PBMCs was subjected to library preparation via hybridization-based target enrichment (Watchmaker Genomics, USA). Sequencing on the Illumina NovaSeq X platform (Illumina, USA) achieved mean coverages of 40× (tumor), 20× (normal), and 500× (target-enhanced panels). Sequence data were aligned to the Genome Reference Consortium Human Build 38 and analyzed via a validated AI-powered bioinformatics pipeline (version 1.4.4; Inocras Inc.) to identify single nucleotide variants, insertions/deletions, copy number alterations, and structural variants. Driver mutations were classified on the basis of the COSMIC database and OncoKB annotations. WGS data for PMGC organoids were obtained from SICR, Yonsei University College of Medicine [40]. Genomic alterations in genes recurrently mutated in GC, on the basis of The Cancer Genome Atlas (TCGA) incidence, were analyzed and visualized [41].

Human leukocyte antigen (HLA) typing was performed via in-house targeted deep sequencing (CancerMaster) of classical class I loci (HLA-A, -B, and -C).

### Cell lines

To provide internal controls for PD-L1 expression analysis, we utilized established breast cancer cell lines (MCF7 and MDA-MB-231) with well-characterized PD-L1 profiles [42–44]. They were purchased from the American Type Culture Collection and cultured in Eagle’s Minimum Essential Medium or RPMI-1640 medium supplemented with 10% fetal bovine serum (Lonza Inc., Basel, Switzerland), 100 units/mL penicillin, and 100 mg/mL streptomycin (Lonza Inc.). The cultured cells were incubated at 37 °C in a humidified atmosphere with 5% CO2.

### Flow cytometry analysis

For PD-L1 analysis, organoids were dissociated with 0.25% trypsin-EDTA (Gibco, Thermo Fisher Scientific, Waltham, MA, USA) and washed with FACS buffer (BioLegend, Inc., San Diego, CA, USA). The cells (5 × 105) were stained with PE-conjugated anti-human CD274 (B7 homolog 1 and PD-L1) (#329705; BioLegend, Inc.) and a matched isotype control (#400311; BioLegend, Inc.).

Flow cytometry analysis was performed on pooled PBMCs to characterize lymphocyte subsets and assess T-cell activation. A total of 5 × 105 cells were stained with the following fluorochrome-conjugated antibodies: FITC-conjugated anti-human CD3 (#300405; BioLegend, Inc.), PE-conjugated anti-human CD4 (#300507; BioLegend, Inc.), PE/cyanine7-conjugated anti-human CD8/-344711; BioLegend, Inc.), PerCP/cyanine5.5-conjugated anti-human CD56 (NCAM) (#304625; BioLegend, Inc.), and APC-conjugated anti-human CD25 (#302609; BioLegend). The matched isotype controls were PerCP/cyanine5.5 mouse IgG2a (κ, #400251; BioLegend, Inc.) and APC mouse IgG1 (κ, #400119; BioLegend, Inc.). The gating strategy is detailed in Additional file 1: Fig. S1.

To assess PD-1 expression in lymphocytes, samples were collected at three time points, immediately after thawing, after 24 h of culture, and following 48 h of CD3/CD28 stimulation, and the samples were stained with APC-conjugated anti-human CD279 (PD-1) (#329907; BioLegend, Inc.) and APC-conjugated mouse IgG1, κ isotype control (#400119; BioLegend, Inc.). The lymphocytes were subsequently gated on forward/side scatter parameters before PD-1 expression analysis.

After staining in each assay, the cells were washed twice, suspended in 500 µL of FACS buffer (BioLegend, Inc.), and acquired via BD Symphony A5 (BD Biosciences, San Jose, CA, USA). The data were analyzed via FlowJo software (version 10.10.0; Tree Star, Ashland, OR, USA).

### PBMC isolation and pooling

PBMCs were isolated from four healthy adult donors (two in their 20s and two in their 50–60 s) to create a diverse immune pool that better represents the immune repertoire of the general population and potentially mitigates donor-specific biases. PBMCs were isolated via a Ficoll density gradient (900 × *g*, 30 min, 20 °C) and washed with PBS. Equal numbers of cells from each donor were pooled and cryopreserved in CELLBANKER 2 medium (ZENOAQ; Fukushima, Japan).

### PBMC culture and activation

Pooled PBMCs were cultured in RPMI-1640 medium containing 10% fetal bovine serum (Lonza, Inc.), 100 units/mL penicillin, and 100 mg/mL streptomycin (Lonza, Inc.), hereafter referred to as T-cell medium. The cultured cells were incubated at 37 °C in a humidified atmosphere with 5% CO2. For T-cell activation, PBMCs were stimulated with Dynabeads Human T-Activator CD3/CD28 (Thermo Fisher Scientific) at a bead-to-cell ratio of 1:1 for 48 h, following the manufacturer’s protocol.

### Establishment of a PMGC organoid and pooled PBMC co-culture model

PMGC organoids were cultured in growth factor-reduced Matrigel domes until they reached a diameter of approximately 50 μm. For imaging-based optimization of co-culture conditions, preactivated PBMCs (stimulated with CD3/CD28 Dynabeads, as described above) were labeled with CellTrace Far Red (#C34564; Thermo Fisher Scientific). To optimize the co-culture conditions, organoids and labeled PBMCs were combined at a 200:1 ratio via two approaches: (1) the suspension method, in which PBMCs were added in suspension to T-cell medium surrounding preformed Matrigel domes; and (2) the embedded method, in which PBMCs were mixed with organoid cells in Matrigel before dome formation. Co-cultures were maintained in T-cell medium at 37 °C with 5% CO₂ for 72 h. Bright-field and fluorescence images were acquired at 0 and 72 h via an Operetta CLS high-content imaging system (Revvity Inc., Waltham, MA, USA). The relative distribution of PBMCs inside and outside the Matrigel domes was quantified by measuring the total fluorescence intensity within and outside the dome region via ImageJ software (National Institutes of Health, Bethesda, MD, USA).

Effector-to‐target (E: T) ratio-dependent organoid susceptibility was assessed by coculturing activated PBMCs with organoids in Matrigel domes at E: T ratios ranging from 1:1 to 200:1 for 48 h, followed by viability measurement via the Cell Counting Kit-8 (CCK-8; Dojindo, Kumamoto, Japan). CellTrace Far Red labeling was not applied in these viability assays. PBMC-only control wells were included in all co-culture viability assays, and the PBMC-derived background signal was taken into account when interpreting the CCK-8 readout. Organoids exhibiting < 50% viability at an E: T ratio of 200:1 were classified as cytolytic, whereas those retaining ≥ 50% viability were classified as noncytolytic.

### Assessment of the effects of Dynabeads on PMGC organoids

To assess the potential toxicity of CD3/CD28 Dynabeads, organoids were embedded in Matrigel with or without CD3/CD28 Dynabeads and cultured for 72 h. Cell viability was measured via a CCK-8 (Dojindo) following the manufacturer’s protocols.

### Co-culture medium optimization

To determine the optimal medium for co-culture, organoids and PBMCs were separately embedded in 4 µL of Matrigel domes and cultured for 72 h in either organoid or T-cell medium. Morphology, PBMC retention, and organoid structural integrity were assessed at 0 and 72 h via bright-field microscopy on an Operetta CLS high-content imaging system (Revvity, Inc.). Organoid viability was measured at 72 h via the CCK-8 assay (Dojindo).

### Proteomic analysis

Proteomic data for PMGC organoids were obtained from the genome database of SICR, Yonsei University College of Medicine [15, 40]. To compare the noncytolytic and cytolytic organoid groups, differential expression analysis was performed via DESeq2 (Bioconductor, R package v1.40.0), with proteins with a |fold change| ≥ 2 and a false discovery rate (FDR) < 0.1 considered significant. Supervised hierarchical clustering was performed via Euclidean distance and complete linkage methods. Gene set enrichment analysis (GSEA) was performed via GSEA software (version 4.3.2; Broad Institute, Cambridge, MA, USA) via the Molecular Signatures Database hallmark gene sets. The protein expression of the coinhibitory ligands FGL1 (ligand for lymphocyte-activation gene 3 [LAG-3]), CD155 and CD112 (ligands for T-cell immunoreceptor with Ig and ITIM domains [TIGIT]), and GAL9 (ligand for T-cell immunoglobulin and mucin-domain containing-3 [TIM-3]) was quantified via normalized expression values.

### PD-1 blockaded test in co-culture

Pembrolizumab concentrations (107 and 256 µg/mL) were selected to reflect the clinical Cₘₐₓ observed at 3 and 10 mg/kg doses in first-in-human studies [45], ensuring translational relevance. PBMCs were activated as described above in the presence of pembrolizumab (107 and 256 µg/mL) for 48 h before co-culture. PBMC viability under monoculture confirmed that there was no direct drug toxicity, as measured by the CCK-8 assay (Dojindo). Subsequently, treated or untreated PBMCs were co-cultured with PD-L1-high PMGC organoids at an E: T ratio of 50:1 for 48 h. Organoid viability was measured via the CCK-8 assay (Dojindo) at the end of the co-culture and normalized to that of the control co-cultures with untreated PBMCs.

### Time-lapse imaging

To monitor PBMC motility and recruitment into PMGC organoids, CellTrace Far Red-labeled PBMCs were co-cultured with organoids embedded in Matrigel domes and imaged via the Operetta CLS high-content imaging system (Revvity, Inc.). Time-lapse images were acquired every 5 min over a 100-min period in Matrigel. The image resolution was 1,920 × 1,440 pixels. Images were analyzed via Harmony software (version 4.9; Revvity, Inc.).

### Statistical analysis

The data are presented as the means ± standard deviations. Paired comparisons were performed via Student’s *t* test (ns: not significant; *p < 0.05; **p < 0.01; and ***p < 0.001). Analyses were performed via SPSS software (version 28).

## Results

### Establishment and characterization of PMGC organoids and activated PBMCs

**Fig. 1:**
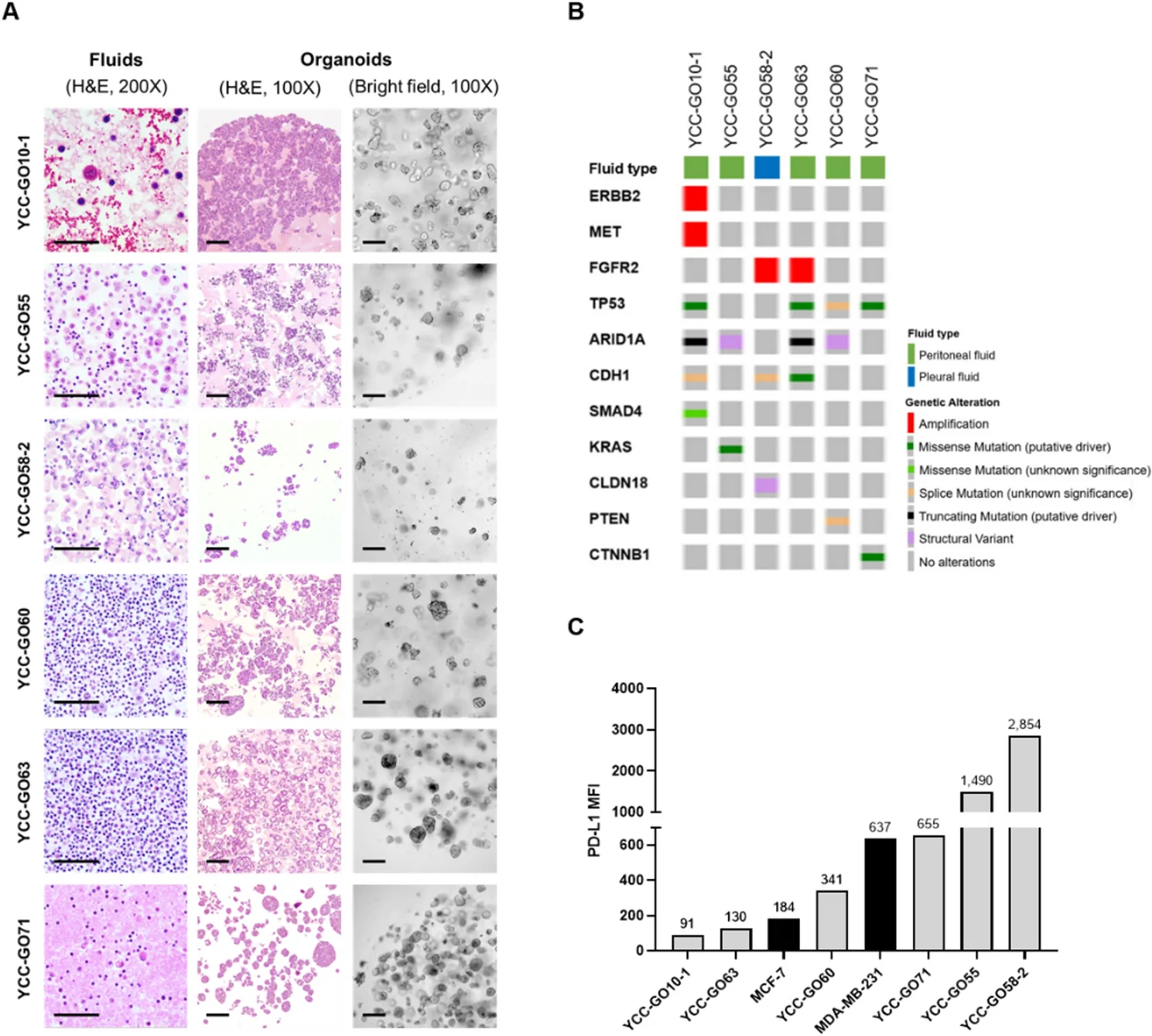
Organoids are generated from the peritoneal or pleural fluid of patients with peritoneal metastatic gastric cancer. (**A**) Representative images of H&E-stained tumor cells from ascites or pleural fluid (200×, left), H&E-stained organoids (100×, middle), and bright-field microscopy images of the organoids (100×, right). (**B**) Genomic profiling of organoids. Whole-genome sequencing of six organoid lines revealed alterations in genes from the 30-gene panel recurrently altered in gastric cancer, including receptor tyrosine kinase pathway genes, as reported in The Cancer Genome Atlas [45]. The full panel is illustrated in Additional file 1: Fig. S2. Columns represent individual organoid lines; rows list only those genes from that panel with detectable alterations in our organoid lines; fluid type is indicated by green (peritoneal) or blue (pleural) bars above each column. (**C**) Programmed death-ligand 1 expression levels were measured via flow cytometry and quantified as the mean fluorescence intensity. The gray bars indicate organoids; the black bars represent the control cell lines, MDA-MB-231 (high), and MCF-7 (negative control)

Organoids were successfully established from malignant peritoneal and pleural effusions collected from patients with peritoneal metastatic gastric cancer (PMGC). These organoid lines demonstrated stable growth for over five passages, reflecting the heterogeneity of PMGC, and the derived organoids retained diverse histological subtypes and HER2/PD-L1 statuses (Table 1). Histological evaluation of the cell pellets and corresponding organoid sections confirmed the presence of malignant cells. Bright-field imaging revealed interline morphological heterogeneity, including loose grape-like clusters (YCC-GO55), well-defined glandular structures (YCC-GO63), and compact spheroids without prominent luminal features (Fig. 1A).

Target-enhanced whole-genome sequencing across 30 recurrently altered gastric cancer genes revealed diverse genomic alterations in the organoid panel (Fig. 1B; Additional file 1: Fig. S2). ERBB2 and MET coamplification was observed exclusively in YCC-GO10-1, whereas FGFR2 amplification was detected in YCC-GO58-2 and YCC-GO60. Additional alterations included TP53, ARID1A, CDH1, and KRAS variants, which is consistent with the known mutational spectrum of gastric cancer.

Surface PD-L1 expression varied substantially across the six organoid models. Flow cytometric analysis classified YCC-GO71, YCC-GO55, and YCC-GO58-2 as PD-L1-high, YCC-GO60 as moderate, and YCC-GO10-1 and YCC-GO63 as low, with MDA-MB-231 and MCF-7 cells used as reference controls (Fig. 1C).

To generate immune effector cells for co-culture assays, pooled PBMCs from four healthy donors were thawed, rested, and stimulated with CD3/CD28 Dynabeads. The number of viable cells increased modestly following stimulation, whereas the number of unstimulated PBMCs decreased over the same period (Additional file 1: Fig. S3Aand B). Flow cytometry revealed increases in CD4⁺, CD8⁺, and NKT cell fractions following stimulation, accompanied by marked expansion of activated T-cell subsets within the CD4⁺ and CD8⁺ populations (Additional file 1: Fig. S3C and D). Cytokine profiling further confirmed immune activation, with elevations in IFN-γ, TNF, IL-6, and IL-2 relative to those in unstimulated controls (Additional file 1: Fig. S3E). PD-1 expression on lymphocytes increased after stimulation (Additional file 1: Fig. S3F), supporting the suitability of these cells for subsequent immune-organoid assay development.

**Table 1:** Clinical and molecular characterization of patients with gastric cancer

	YCC- GO10-1	YCC- GO55	YCC- GO58-2	YCC- GO60	YCC- GO63	YCC- GO71
**Age at diagnosis**	36	32	40	72	31	44
**Sex**	Female	Male	Male	Female	Female	Male
**Stage**	Ⅳ	Ⅳ	Ⅳ	Ⅳ	Ⅳ	Ⅳ
**Histology** ^*^	APD	SRC/PCC	AMD	SRC/PCC	SRC/PCC	APD
			+SRC/PCC			
**Lauren classification**	Unknown	Unknown	Unknown	Unknown	Unknown	Unknown
**Interval between** **collection to death** (months)**	4.40	0.93	0.57	0.37	6.27	0.70
**HER2**	Positive	Negative	Negative	Negative	Negative	Positive
**EBV**	Negative	Negative	Negative	Negative	Negative	Positive
**PD-L1 CPS**	0	0	< 1	< 1	0	5

All patients had previously received at least two lines of chemotherapy, and tumor cells were detected in all collected fluid samples. *APD, adenocarcinoma/poorly differentiated; AMP, adenocarcinoma/moderately differentiated; SRC, signet ring cell type; PCC, poorly cohesive carcinoma; all patients have proficient mismatch repair. **Interval between fluid collection and death is calculated as (Date of death - Date of fluid collection)/30. HER2 status was determined by immunohistochemistry (IHC) using an anti-HER2 4B5 antibody (Ventana Medical Systems, Tucson, AZ, USA) following the ASCO/CAP guidelines. Epstein-Barr virus (EBV) status is assessed by in situ hybridization of EBV-encoded small RNA via the Ventana Benchmark ISH system and ISH iView kit. (Ventana Medical Systems). PD-L1 expression was assessed via a PD-L1 IHC 22C3 pharmDx assay (Agilent Technologies/Dako, Carpinteria, CA, USA) with combined positive score (CPS) calculation

### Optimization of 3D co-culture conditions for PBMC-organoid interactions

To establish a stable co-culture environment enabling prolonged contact between immune cells and organoids, both the culture medium and spatial configuration were evaluated. Organoid structural integrity was maintained in both organoid and T-cell media; however, PBMCs remained confined within the Matrigel dome only in T-cell medium, whereas they gradually migrated outside the dome in organoid medium (Additional file 1: Fig. S4A-C). Accordingly, T-cell medium was selected for subsequent experiments.

**Fig. 2:**
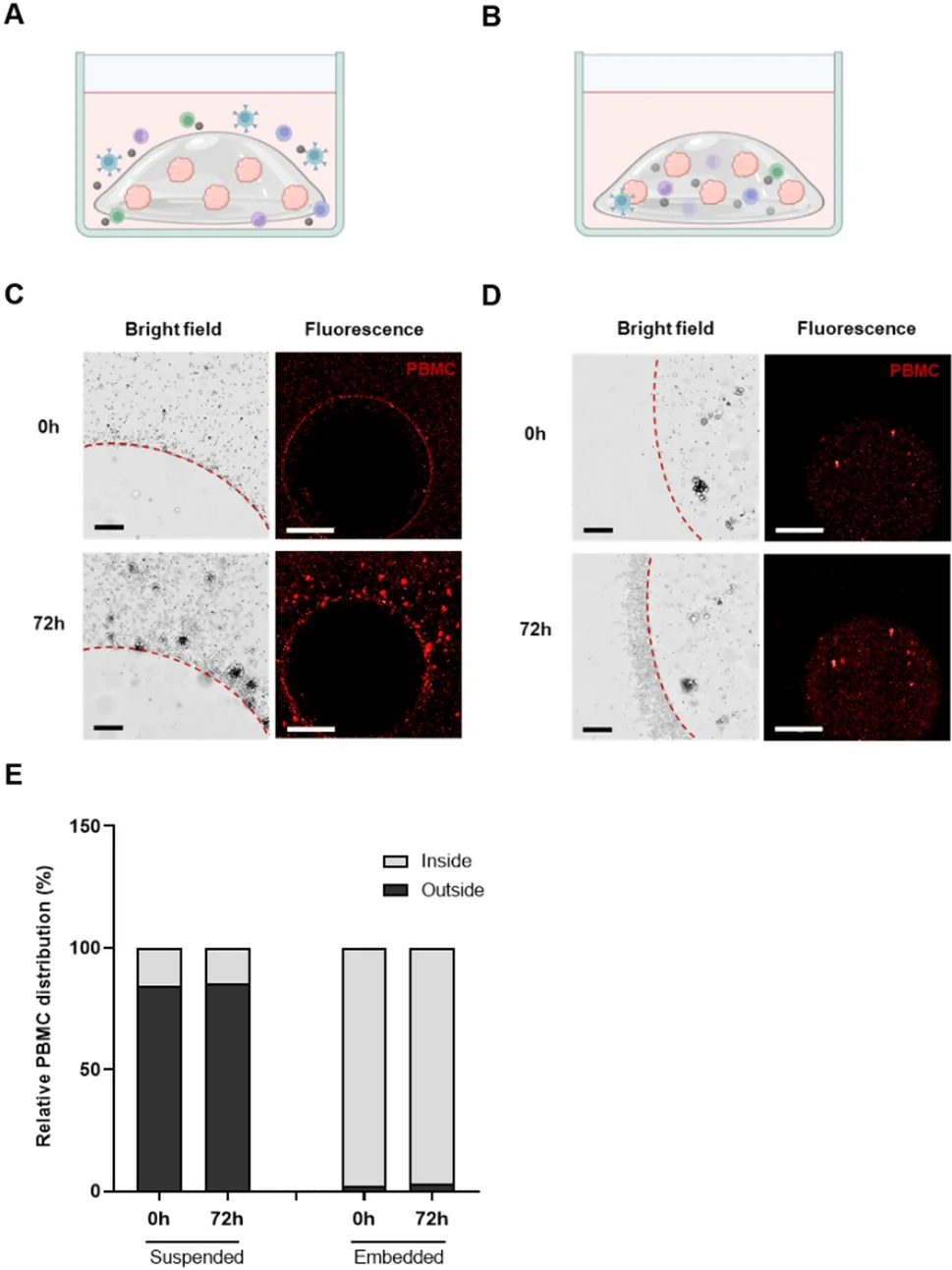
Establishment of peritoneal metastatic gastric cancer organoids and pooled PBMC co-culture model. Two co-culture configurations were compared to determine the optimal setup for modeling tumor-immune interactions. (**A**-**B**) Schematic illustrations of the two co-culture configurations. CellTrace Far Red-labeled PBMCs were added either (**A**) suspended in culture medium outside Matrigel domes or (**B**) embedded within Matrigel domes containing gastric cancer organoids. Figs were created via BioRender. (**C**-**D**) Representative bright-field and fluorescence microscopy images of co-culture configurations at 0 and 72 h. (**C**) Suspended co-culture demonstrating that PBMCs remain predominantly outside the dome (the red dashed line indicates the dome boundary). (**D**) Embedded co-culture demonstrating PBMC retention within the dome. Black scale bars, 50 μm. White scale bars, 500 μm. (**E**) Bars indicate the proportion of the PBMC signal detected inside or outside the dome for 72 h, normalized to the total red intensity

Two spatial configurations, suspended and embedded, were compared via CellTrace-labeled activated PBMCs. In the suspended setup, PBMCs were added atop solidified Matrigel domes and largely remained outside the dome at 72 h with minimal infiltration (Fig. 2A and C). In contrast, the embedded configuration, in which PBMCs and organoids were co-embedded in Matrigel, resulted in sustained PBMC localization within the dome (Fig. 2B and D). Quantitative fluorescence measurements, normalized to the Matrigel region containing organoids, confirmed substantially greater PBMC signal retention in the embedded condition at 0 h and 72 h (Fig. 2E). Organoid viability was unaffected by the presence of CD3/CD28 beads under embedded conditions (Additional file 1: Fig. S5A and B).

### Embedded co-culture supports dynamic PBMC infiltration and heterogeneous cytotoxic responses

**Fig. 3:**
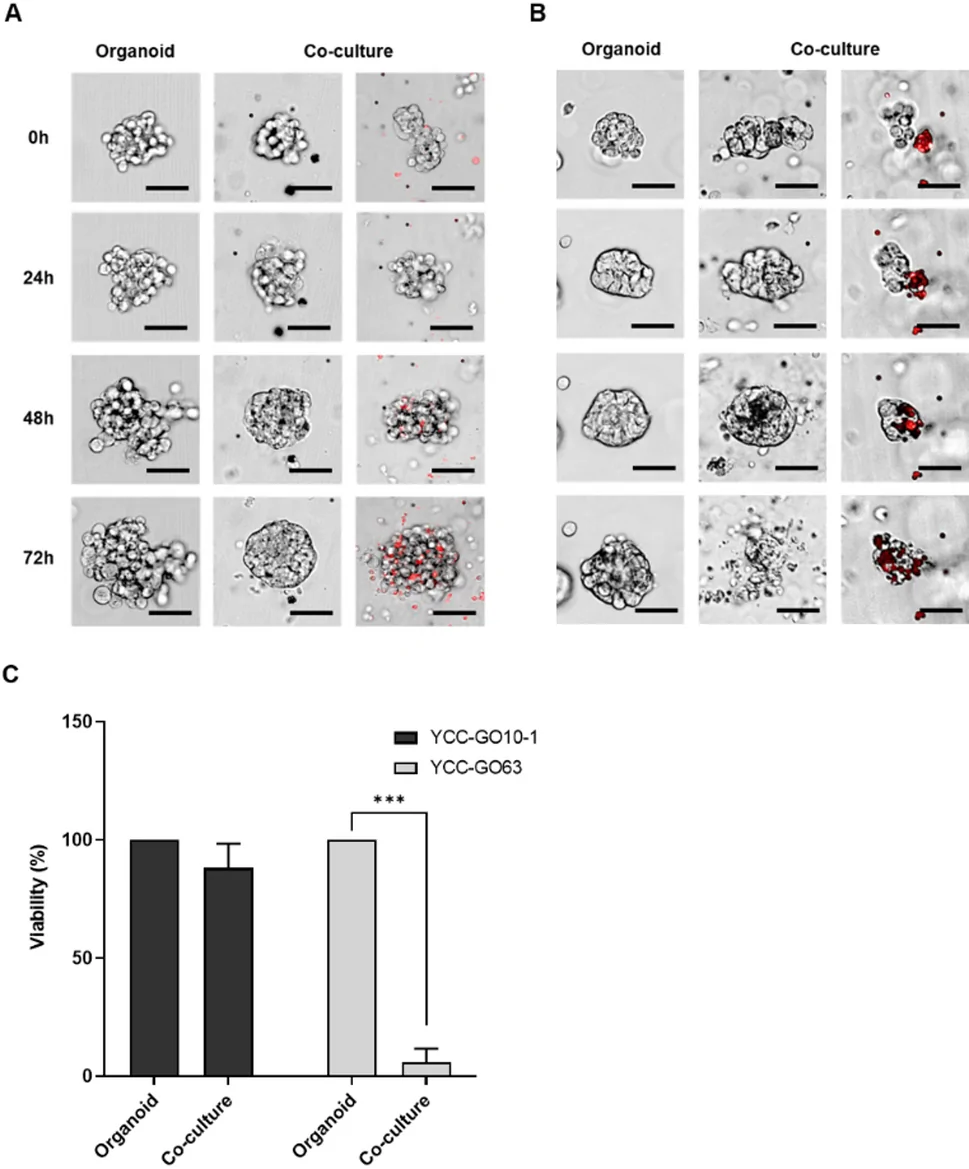
The embedded co-culture configuration enables dynamic PBMC-organoid interactions and demonstrates organoid-specific cytotoxic responses. Time-lapse bright-field microscopy demonstrating PBMC migration and organoid-PBMC interactions in the embedded co-culture. Representative images of (**A**) YCC-GO10-1 and (**B**) YCC-GO63 organoids co-cultured with CellTrace Far Red-labeled PBMCs for 72 h. Red signals indicate PBMC localization, demonstrating progressive PBMC migration toward and around organoids within Matrigel. Left row: organoids cultured without PBMCs; middle and right rows: organoids co-cultured with pooled PBMCs; scale bar: 50 μm. (**C**) Organoid viability following 72 h of co-culture was assessed via the Cell Counting Kit-8 assay and normalized to that of organoid-only controls. The data revealed line-specific immune sensitivity, with YCC-GO63 demonstrating significant cytotoxicity compared with YCC-GO10-1 (***p < 0.001). The error bars represent the means ± standard errors of the means; *n* ≥ 3 independent experiments

Time-lapse imaging revealed that activated PBMCs actively migrated within Matrigel and infiltrated organoid structures over 72 h, whereas unstimulated PBMCs exhibited limited mobility (Additional file 2 and Additional file 3). Distinct immune susceptibility patterns were observed among the organoid lines. YCC-GO10-1 retained intact epithelial architecture throughout co-culture, whereas YCC-GO63 progressively lost structural integrity (Fig. 3A and B). Quantitative viability analysis after 72 h of co-culture, normalized to that of the untreated controls, indicated minimal cytotoxicity in YCC-GO10-1 cells and a significant reduction in viability in YCC-GO63 cells (***, *p* < 0.001; Fig. 3C).

### Heterogeneous cytotoxicity differentiates cytolytic and noncytolytic organoid phenotypes

**Fig. 4:**
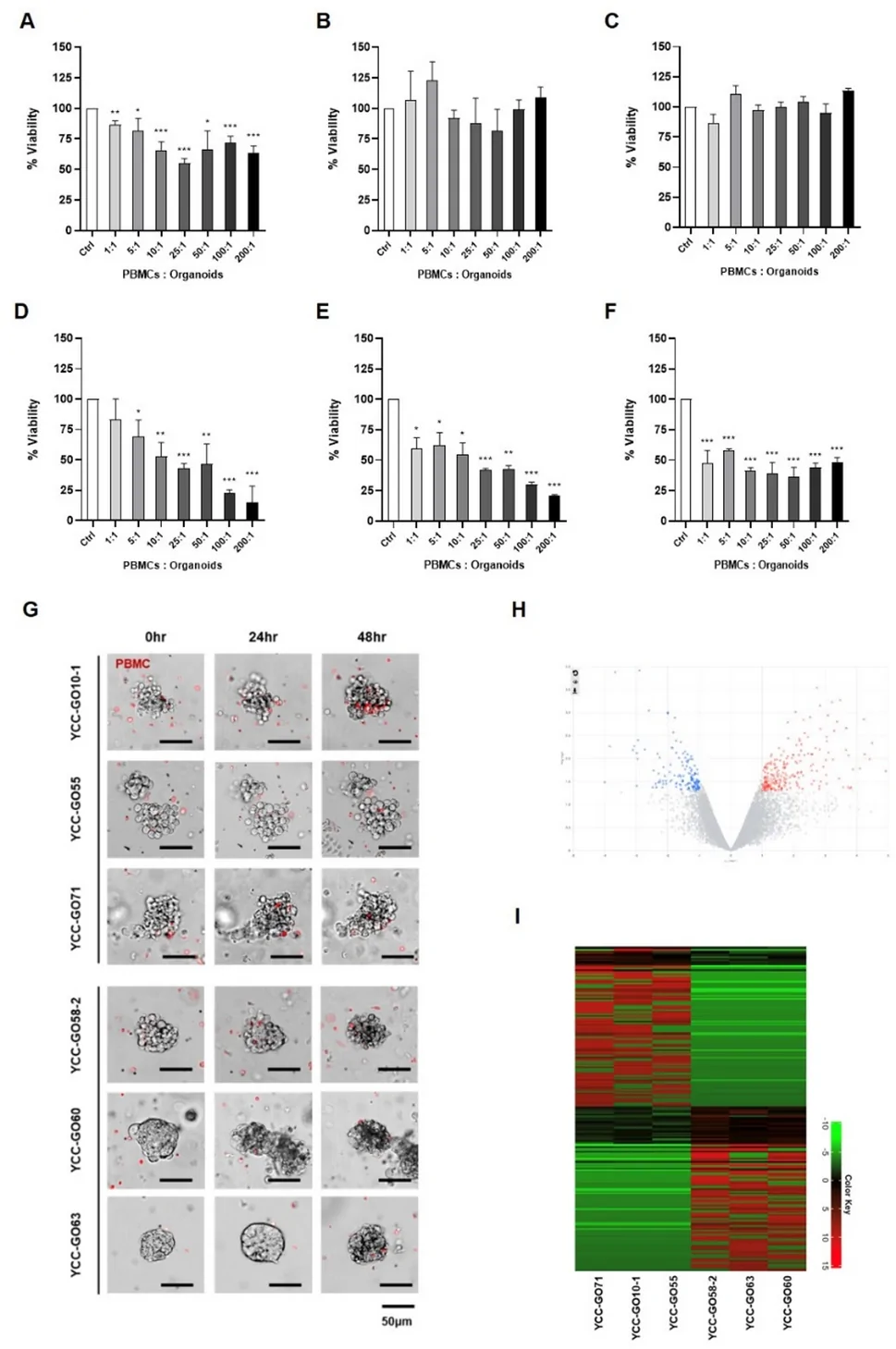
Immune susceptibility of peritoneal metastatic gastric cancer organoids to PBMC-mediated cytotoxicity. (**A**-**F**) Organoid viability was measured via the Cell Counting Kit-8 assay following 48 h of co-culture with activated pooled PBMCs at varying effector-to-target (E: T) ratios (1:1 to 200:1). (**A**) YCC-GO10-1, (**B**) YCC-GO55, and (**C**) YCC-GO71 demonstrate noncytolytic responses, whereas (**D**) YCC-GO63, (**E**) YCC-GO60, and (**F**) YCC-GO58-2 demonstrate cytotoxic susceptibility. The data are presented as the means ± standard errors of the means (*n* = 3 independent experiments). (**G**) Representative time-lapse merged bright-field and fluorescence images of embedded organoid-PBMC co-cultures at an E: T ratio of 200:1 over 48 h. The top three organoid lines retained intact morphology with minimal structural alterations in the bright field, whereas the bottom three lines demonstrated progressive darkening and collapse, which was consistent with PBMC-induced cell death. Red fluorescence indicates CellTrace Far Red-labeled PBMCs. Scale bars, 50 μm. (**H**) Differential protein expression between cytolytic and noncytolytic organoid lines was determined via DESeq2 (|log₂-fold change| ≥ 2, FDR < 0.1). The volcano plot displays the -log₁₀ adjusted p value versus the log₂ fold change, highlighting 115 upregulated (red) and 112 downregulated (blue) proteins in nonresponsiveorganoids. (**I**) Supervised hierarchical clustering is performed via Euclidean distance and complete linkage methods. The heatmap shows the hierarchical clustering of differentially expressed proteins, demonstrating distinct expression profiles between cytolytic and noncytolytic organoid lines

To further assess immune susceptibility, organoids were co-cultured with activated PBMCs at effector-to-target (E: T) ratios ranging from 1:1 to 200:1 for 48 h. Three organoid lines, YCC-GO10-1, YCC-GO55, and YCC-GO71, retained > 50% viability across conditions and preserved spheroid architecture and were categorized as noncytolytic models (Fig. 4A and C). In contrast, YCC-GO58-2, YCC-GO60, and YCC-GO63 displayed dose-dependent loss of viability accompanied by morphological disruption, which was consistent with the observed cytolytic phenotypes (Fig. 4D and F). Representative images at E: T = 200:1 illustrate the contrast between intact spheroids (noncytolytic lines) and disrupted structures (cytolytic lines) (Fig. 4G).

HLA typing of organoids and PBMC donors revealed diverse alleles across samples (Table 2). No clear relationship was observed between shared HLA allotypes and cytolytic susceptibility, indicating that other tumor-intrinsic factors may contribute to the differential responses.

Comparative proteomic profiling revealed proteins that were differentially expressed between cytolytic and noncytolytic organoids (|log₂FC| ≥ 2, FDR < 0.1), with 115 proteins enriched in noncytolytic lines and 112 enriched in cytolytic lines (Fig. 4H and Additional file 4). Supervised clustering segregated the organoid lines in accordance with their susceptibility classification (Fig. 4I). GSEA revealed that noncytolytic organoids were significantly enriched for the NK cell-mediated cytotoxicity pathway (normalized enrichment score [NES] = 2.1, q < 0.01), whereas cytolytic organoids were enriched for metabolic pathways (NES = -2.3, q < 0.01), drug metabolism-cytochrome P450 (NES = -1.9, FDR q < 0.05), and linoleic acid metabolism (NES = -1.8, q < 0.05) (Additional file 5). Pathway enrichment analysis revealed differences in metabolic and immune-related programs; however, these findings are exploratory and require external validation.

**Table 2:** HLA-type PBMCs from PMGC organoids and healthy donors

Sample		HLA-A		HLA-B		HLA-C	
**Organoid**	**YCC-GO10-1**	A*02:01	A*02:01	B*48:01	B*51:01	C*14:02	C*14:02
	**YCC-GO55**	A*03:02	A*24:02	B*40:02	B*51:01	C*03:04	C*14
	**YCC-GO58-2**	A*11:01	A*11:01	B*40	B*40:01	C*04:01	C*04:82
	**YCC-GO60**	A*02:07	A*11:01	B*46:01	B*51:01	C*01:02	C*14:02
	**YCC-GO63**	A*11:01	A*11:01	B*40:01	B*40:01	C*07:02	C*07:66
	**YCC-GO71**	A*26:01	A*33:03	B*40:01	B*58	C*03:02	C*04:82
**PBMC**	**Donor 1**	A*11:04	A*11:04	B*35:43	B*51:02	C*04:03	C*14:03
	**Donor 2**	A*02:79	A*26:01	B*40:02	B*40:02	C*01:14	C*08:24
	**Donor 3**	A*11:04	A*31:01	B*51:02	B*44:03	C*14:02	C*14:09
	**Donor 4**	A*02:09	A*02:09	B*15:08	B*48:03	C*07:149	C*08:01

### Application of the platform to evaluate the PD-1 blockade response

**Fig. 5:**
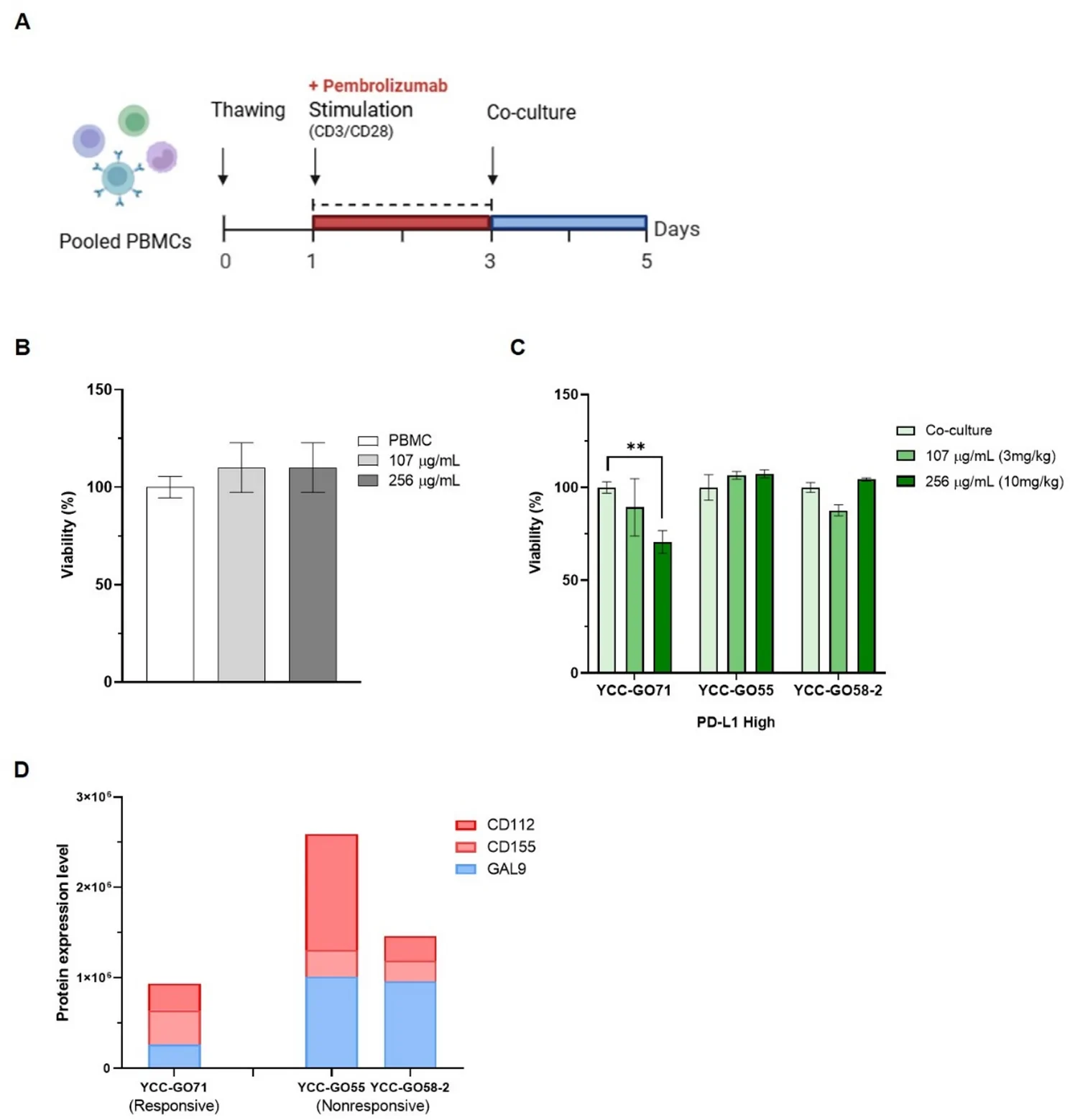
PD-1 blockade responses in PD-L1-high organoids and the baseline expression of alternative coinhibitory ligands. (**A**) Schematic illustration of the PD-1 blockade test. Pooled PBMCs were thawed and stabilized for 24 h and then activated with CD3/CD28 Dynabeads in the presence of pembrolizumab for 48 h, followed by co-culture with PD-L1-high organoids at an effector-to-target ratio of 50:1 for 48 h. The figure was created via BioRender. (**B**) PBMC viability under monoculture conditions after 48 h of exposure to pembrolizumab was measured via the CCK-8 assay and normalized to that of untreated PBMC controls. No significant cytotoxicity was observed at either antibody concentration. The data are presented as the means ± standard deviations (SDs) of three independent experiments. (**C**) Organoid viability following 48 h of co-culture with PD-1 inhibitor-treated PBMCs was assessed via the CCK-8 assay and normalized to that following co-culture with untreated PBMCs. Among the PD-L1-high organoid lines, only YCC-GO71 exhibited a significant reduction in viability with pembrolizumab at 256 µg/mL (**p < 0.01), whereas YCC-GO55 and YCC-GO58-2 remained nonresponsive. The data are presented as the means ± SDs of three independent experiments. Statistical analysis was performed via an unpaired two-tailed Student’s t test. **p < 0.01. (**D**) Proteomic analysis of coinhibitory ligand expression in PD-L1-high organoids. The baseline protein expression levels of CD155 and CD112 (T-cell immunoreceptor with Ig and ITIM domain ligands) and galectin-9 (T-cell immunoglobulin and mucin-domain containing-3 ligand) are shown for YCC-GO71 (responsive), YCC-GO55, and YCC-GO58-2 (nonresponsive)

To examine the utility of the platform for assessing immune checkpoint modulation, PD-L1-high organoids were co-cultured with activated PBMCs pretreated with pembrolizumab at clinically relevant concentrations (Fig. 5A). Pembrolizumab did not affect PBMC viability in monocultures (Fig. 5B). Among the three PD-L1-high organoid types, YCC-GO71 exhibited increased cytotoxicity upon pembrolizumab treatment at 256 µg/mL, whereas YCC-GO55 and YCC-GO58-2 did not show a detectable increase in cytotoxicity (Fig. 5C). Similarly, the PD-L1-moderate and PD-L1-low lines did not exhibit increased cytotoxicity (Additional file 1: Fig. S6 A and B).

Proteomic analysis revealed higher baseline expression of several alternative immune checkpoint ligands, including CD112 (TIGIT ligand) and galectin-9 (TIM-3 ligand), in the two nonresponsive organoid lines than in the YCC-GO71 line (Fig. 5D). These findings identify molecular differences associated with differential responses to PD-1 blockade; however, these observations are descriptive and require further functional validation.

## Discussion

In this study, we established a standardized 3D co-culture platform integrating patient-derived organoids from peritoneal metastatic gastric cancer (PMGC) with activated peripheral blood mononuclear cells (PBMCs) to functionally assess tumor-immune interactions and susceptibility to immune-mediated cytotoxicity. By optimizing the spatial configuration and culture conditions to maintain the proximity and viability of both components, this system enabled dynamic infiltration of activated T cells into organoid structures and revealed heterogeneous cytolytic responses across PMGC organoid lines. These results highlight the intrinsic diversity of immune susceptibility in PMGCs and demonstrate the utility of organoid-based functional assays for exploring tumor-immune dynamics. Pooled PBMCs from multiple healthy donors ensured sufficient immune cell numbers and reduced the degree of dependence on single-donor or autologous samples.

Our data demonstrate that preactivated PBMCs can dynamically infiltrate Matrigel-embedded organoids, enabling real-time visualization of tumor-immune cell interactions and quantification of organoid-specific cytotoxicity. The observed heterogeneity in immune susceptibility across the six PMGC organoid lines mirrors the clinical variability in immunotherapy responses and highlights the limitations of PD-L1 expression as a predictive biomarker [46, 47].

A key finding of this work is that cytolytic and noncytolytic phenotypes emerged independently of HLA matching between organoids and PBMC donors. Despite complete HLA mismatch in this allogeneic setting, susceptibility patterns are not explained by shared or incompatible HLA alleles, suggesting that tumor-intrinsic factors dominate early immune engagement in this model [48, 49]. The lack of association between cytolytic susceptibility and the degree of HLA mismatch suggests that immune killing in this platform is not determined solely by donor–recipient HLA compatibility. In PBMC-based co-culture systems, cytotoxicity may reflect the activity of multiple effector populations, including activated T cells and NK/NKT-cell populations, rather than a single HLA-restricted mechanism. Although our GSEA results showed enrichment of NK cell-mediated cytotoxicity pathways, this finding alone is insufficient to conclude that NK cells were the dominant effectors in the present model. Given that PBMCs were preactivated with anti-CD3/CD28 and that unstimulated PBMCs showed limited motility, activated T-cell responses likely also contributed to the observed immune interactions. Therefore, the HLA-independent cytotoxicity observed here likely reflects the combined effects of tumor-intrinsic susceptibility and multiple activated immune-cell subsets, and future studies will be needed to define the relative contributions of T cells and NK/NKT-cell populations in this system [50–53]. Comparative proteomic profiling supported this interpretation: cytolytic organoids were enriched for metabolic pathways, including drug metabolism-cytochrome P450 signatures, whereas noncytolytic lines exhibited enrichment of NK-cell-mediated cytotoxicity signatures. Prior studies have shown that tumor-intrinsic metabolic states can influence antitumor immunity by altering nutrient availability, redox balance, lipid handling, and the accumulation of immunomodulatory metabolites within the tumor microenvironment, thereby affecting T-cell fitness, infiltration, and responsiveness to immunotherapy [54–58]. In this context, our findings suggest that distinct tumor-intrinsic molecular programs are associated with differential immune susceptibility in PMGC. However, the functional significance of the metabolic pathways identified in the cytolytic group remains to be determined and warrants further mechanistic investigation. Although these associations require further validation, they provide hypothesis-generating insights into molecular programs associated with susceptibility to immune-mediated killing in PMGC.

The platform also allowed exploratory assessment of PD-1 blockade responsiveness. Notably, only one of the three PD-L1-high organoid lines exhibited increased cytotoxicity following pembrolizumab exposure, whereas the remaining two lines were nonresponsive despite comparable PD-L1 expression. Proteomic analysis indicated that nonresponsive PD-L1-high organoids exhibited a higher baseline expression of alternative immune checkpoint ligands, including CD112 and galectin-9. Prior studies have shown that the upregulation of alternative inhibitory pathways, particularly TIM-3, can accompany adaptive resistance to PD-1/PD-L1 blockade. In this context, the concordance between our observations and previous reports suggests that this co-culture model may capture baseline features associated with PD-1 nonresponse [59–62]. However, we did not directly assess the expression of the corresponding receptors on infiltrating lymphocytes, including TIGIT and TIM-3, after co-culture, and therefore the present data do not establish whether these ligand–receptor axes were functionally engaged in our system. Nevertheless, this observation remains biologically relevant, as both the TIGIT and TIM-3 pathways have emerged as important alternative inhibitory checkpoints associated with immune dysfunction and resistance to PD-1 blockade in solid tumors. In particular, TIGIT expression is closely associated with PD-1 expression and impaired antitumor immunity, whereas TIM-3 has been implicated in compensatory or adaptive resistance during PD-1 blockade. In gastric cancer, the galectin-9/TIM-3 axis has also been proposed as a potential mediator of T-cell dysfunction and anti-PD-1 resistance. Therefore, although our current data do not define the functional status of these receptor–ligand interactions, the elevated expression of CD112 and galectin-9 in nonresponsive organoids provides a rationale for future studies examining checkpoint receptor expression on infiltrating immune cells and for exploring these pathways as candidate resistance mechanisms in this platform [63–65].

Several considerations should be noted when these results are interpreted. First, the use of pooled allogeneic PBMCs introduces complexity related to donor-specific immune heterogeneity and alloreactive interactions. Although the observed susceptibility patterns were not associated with specific donor-tumor HLA combinations, the system does not fully recapitulate antigen-specific T-cell recognition. Second, PBMCs are activated via CD3/CD28 stimulation, which induces broad polyclonal activation rather than physiologic antigen-driven exhaustion states. Consequently, the effects of pembrolizumab observed here reflect the modulation of activated T-cell function rather than the reversal of exhaustion, limiting direct extrapolation to in vivo therapeutic responses. Third, the organoid panel size was modest (*n* = 6), which constrains the statistical power of proteomic comparisons and pathway enrichment analyses. The proteomic signatures identified in this study should therefore be considered exploratory and warrant confirmation in expanded organoid cohorts or clinical samples. Finally, the current model does not incorporate additional tumor microenvironment components, such as cancer-associated fibroblasts, myeloid cells, or stromal architecture, all of which contribute substantially to immune evasion in metastatic gastric cancer [23, 66].

Despite these limitations, the present system provides a tractable and physiologically relevant approach for the functional evaluation of tumor-immune interactions in PMGC. By enabling standardized assessment of immune infiltration, cytotoxicity, and checkpoint-modulated responses, this platform offers a foundation for future mechanistic studies and may support the development of individualized immunotherapy strategies. Integration with stromal components, autologous immune cells, or genetically perturbed organoids will further increase the translational applicability of this platform.

Collectively, our findings present an organoid-based framework for profiling immune susceptibility in PMGCs and provide hypothesis-generating insights into both intrinsic determinants of the cytolytic response and alternative checkpoint-mediated resistance.

## Conclusions

We established an allogeneic 3D pooled PBMC-organoid co-culture platform in which both immune and tumor cells are embedded within a three-dimensional matrix, enabling functional evaluation of tumor-immune interactions in PMGC. Heterogeneous immune susceptibility was observed across organoid lines, with cytolytic and noncytolytic phenotypes identified independently of HLA allotype matching. Variable responses to PD-1 blockade were observed among PD-L1-high organoids, with nonresponsive lines showing higher baseline expression of alternative immune checkpoint ligands, CD112 and galectin-9. Therefore, we believe this platform may be suitable for profiling immune susceptibility and evaluating immunotherapy responses in PMGC, and future incorporation of autologous immune components and additional tumor microenvironment elements will further strengthen its translational applicability.

## Supplementary Information

Below is the link to the electronic supplementary material.

Supplementary Material 1: Additional File 1: Figures S1-S6.

Supplementary Material 2: Additional File 2. Differentially expressed proteins between cytolytic and noncytolytic gastric cancer organoids.

Supplementary Material 3: Additional File 3. Gene Set Enrichment Analysis (GSEA) of proteomic profiles in cytolytic and noncytolytic organoids.

Supplementary Material 4: Additional File 4. Time-lapse imaging of organoid co-culture with unstimulated pooled PBMCs.

Supplementary Material 5: Additional File 5. Time-lapse imaging of organoid co-culture with pre-activated pooled PBMCs.

## Data Availability

The whole genome sequencing data generated and analyzed during the current study are available in the NCBI BioProject repository, https://identifiers.org/bioproject: PRJNA1435821 [67]. The proteomics datasets have been deposited to the ProteomeXchange Consortium via the PRIDE partner repository with the dataset identifier PXD076305 (https://identifiers.org/proteomexchange: PXD076305) [68]. For peer review purposes, the proteomics data can be accessed via the PRIDE website using the following credentials: Username: reviewer_pxd076305@ebi.ac.uk, Password: qKSrnNQdep8T.

## Abbreviations

PMGC: peritoneal metastatic gastric cancer
PD-1: programmed death-1
PD-L1: programmed death-ligand 1
GC: gastric cancer
PBMC: peripheral blood mononuclear cell
CD3: cluster of differentiation 3
2D: two-dimensional
3D: three-dimensional
ICIs: immune checkpoint inhibitors
EBV: Epstein-Barr virus
PDOs: patient-derived organoid
HER2: human epidermal growth factor receptor 2
CPS: combined positive score
SICR: Song-Dang Institute for Cancer Research
PBS: phosphate-buffered saline
WGS: whole-genome sequencing
TCGA: The Cancer Genome Atlas
HLA: human leukocyte antigen
RPMI: Roswell Park Memorial Institute
PE: phycoerythrin
PerCP: peridinin-chlorophyll-protein complex
APC: allophycocyanin
FACS: fluorescence-activated cell sorting
E:T: effector‐to‐target
CCK-8: Cell Counting Kit-8
FDR: false discovery rate
GSEA: gene set enrichment analysis
FGL1: fibrinogen-like protein 1
LAG-3: lymphocyte-activation gene 3
TIGIT: T-cell immunoreceptor with Ig and ITIM domains
GAL9: galectin-9
