# Supplementary material for: A 3D pooled PBMC-organoid co-culture platform for profiling immune susceptibility and PD-1 blockade response in gastric cancer
Source: BMC Med. 2026 Jun 24;24:481. doi: 10.1186/s12916-026-05006-4 (PMC13560153; doi:10.1186/s12916-026-05006-4)
Supplement: Supplementary file 1 [file 12916_2026_5006_MOESM1_ESM.docx]

**Additional File 1**

FigS1. Gating strategy for the identification of immune cell subsets in pooled peripheral blood mononuclear cells.

FigS2. Extended genetic profiling of peritoneal metastatic gastric cancer organoids (related to Figure 1B).

FigS3. Characteristics of pooled peripheral blood mononuclear cells (PBMCs) used for co-culture experiments.

FigS4. Effect of co-culture media on organoids and peripheral blood mononuclear cells (PBMCs).

FigS5. Effect of CD3/CD28 Dynabeads on peritoneal metastatic gastric cancer organoids in an embedded configuration.

FigS6. Effect of programmed death-1 (PD-1) blockade in programmed death-ligand 1 (PD-L1) moderate or low organoids.

**FigS1. Gating strategy for the identification of immune cell subsets in pooled peripheral blood mononuclear cells.** Representative flow cytometry plots sequential gating for lymphocyte population analysis. Lymphocytes are gated based on FSC-) and properties, followed by singlet selection using FSC-H vs. FSC-A. CD3^+^ T cells are identified from the lymphocyte population, and further gating distinguished helper T cells (CD3^+^CD4^+^), cytotoxic T cells (CD3^+^CD8^+^), and natural killer (NK) T cells (CD3^+^CD56^+^). NK cells are identified as CD3^–^CD56^+^ lymphocytes.

**FigS2. Extended genetic profiling of peritoneal metastatic gastric cancer organoids (related to Figure 1B).** Whole-genome sequencing of six organoid lines identified alterations in genes from the 30 gene panel recurrently altered in gastric cancer, including receptor tyrosine kinase pathway genes, as reported in The Cancer Genome Atlas [46]. Columns represent individual organoid lines; rows list only those genes from that panel with detectable alterations in our organoid lines; Fluid type is indicated by green (peritoneal) or blue (pleural) bars above each column.

**FigS3. Characteristics of pooled peripheral blood mononuclear cells (PBMCs) used for co-culture experiments.** (A) Schematic overview of pooled PBMC preparation and CD3/CD28 Dynabead-mediated stimulation for generating activated T cells used in co-culture. Pooled PBMCs from four donors are thawed, stabilized for 24 h, and subsequently activated with CD3/CD28 stimulation for 48 h. Figure is created using BioRender. (B) Cell count of pooled PBMC over time with or without stimulation. (C) Composition of lymphocyte subsets (CD4⁺ T cells, CD8⁺ T cells, NKT cells, and NK cells) in pooled PBMCs after 48 h of culture with or without stimulation. (D) Composition of activated T cells (CD25⁺) among CD4⁺ T cells, CD8⁺ T cells, and NKT cells after 48 h of culture with or without stimulation. (E) Concentrations of secreted cytokine (IFN-γ, TNF, IL-6, and IL-2) levels in culture supernatants from pooled PBMCs after 48 h of parallel culture with or without CD3/CD28 stimulation (mean ± standard error of the mean). (F) PD-1 expression in pooled PBMCs at defined time points. Thawed: cells immediately after thawing (Day 0); pre-activated: cells after 24 h of stabilization before CD3/CD28 stimulation (Day 1); and activated: cells after 48 h of CD3/CD28 stimulation (Day 3). Representative histograms and bar graph indicate the percentage of PD-1⁺ cells within the lymphocyte gate at each time point.

**FigS4. Effect of co-culture media on organoids and peripheral blood mononuclear cells (PBMCs).** (A) Representative images of organoids cultured in organoid or T cell medium embedded in Matrigel for 72 h. Scale bars, 50 μm. (B) Representative images of PBMCs cultured in organoid or T cell medium embedded in Matrigel for 72 h. Scale bars, 500 μm. (C) Quantification of PBMC distribution inside or outside the Matrigel dome after 72 h of culture in organoid or T cell medium. Bars indicate the proportion of PBMCs detected inside (light gray) or outside (dark gray) the dome, normalized to total intensity.

**FigS5. Effect of CD3/CD28 Dynabeads on peritoneal metastatic gastric cancer organoids in an embedded configuration.** (A) Representative bright-field images of organoids cultured in Matrigel with or without CD3/CD28 Dynabeads for 72 h. Scale bars, 500 μm. (B) Viability of organoids cultured in Matrigel with or without CD3/CD28 Dynabeads at 72 h. Viability is assessed using CCK-8 assay and expressed as percentage relative to organoid only control. Data are presented as the mean ± standard deviation (n = 3). ns, not significant.

**FigS6. Effect of programmed death-1 (PD-1) blockade in programmed death-ligand 1 (PD-L1) moderate or low organoids.** (A) Organoid viability following 48 h co-culture with PD-1 inhibitor-treated PBMCs is assessed using CCK-8 assay and normalized to co-culture with untreated PBMCs. All PD-L1-moderate or low organoid remained resistant. Data are presented as the mean ± SD of three independent experiments. Statistical analysis is performed using an unpaired two-tailed Student’s t-test. (B) Proteomic analysis of co-inhibitory ligand expression in PD-L1-moderate or low organoids. Protein levels of FGL1 (LAG-3 ligand), CD155/CD112 (TIGIT ligands), and galectin-9 (TIM-3 ligand) are demonstrated for YCC-GO10-1, YCC-GO63, and YCC-GO60 (nonresponsive).

**Additional File 4. Time-lapse imaging of organoid co-culture with pre-activated pooled PBMCs.** Organoids were co-cultured with pre-activated pooled PBMCs at a PBMC-to-organoid ratio of 200:1 for 48 h. Time-lapse images were acquired every five minutes (20 intervals total).

**Additional File 5. Time-lapse imaging of organoid co-culture with unstimulated pooled PBMCs.** Organoids were co-cultured with unstimulated pooled PBMCs at a PBMC-to-organoid ratio of 200:1 for 48 h. Time-lapse images were acquired every five minutes (20 intervals total).
